# Polymyositis with elevated serum IgG4 levels and abundant IgG4^+^ plasma cell infiltration: A case report and literature review

**DOI:** 10.1097/MD.0000000000008710

**Published:** 2017-12-01

**Authors:** Ryusuke Anan, Mitsuhiro Akiyama, Yuko Kaneko, Jun Kikuchi, Kazuko Suzuki, Shiro Matsubara, Tsutomu Takeuchi

**Affiliations:** a Division of Rheumatology, Department of Internal Medicine, Keio University School of Medicine; b Department of Neurology, Tokyo Metropolitan Neurological Hospital, Tokyo, Japan.

**Keywords:** diagnosis, IgG4, IgG4-related disease, plasma cells, polymyositis

## Abstract

**Introduction:**

Polymyositis (PM) is a type of autoimmune, inflammatory myopathy. IgG4-related disease (IgG4-RD) is a recently recognized disease entity characterized by elevated serum IgG4 levels and IgG4^+^ plasma-cell infiltration of various organs. However, several reports have described cases of other diseases that present with those features, suggesting the importance of careful differential diagnosis. Herein, we report the first case of PM with elevated serum IgG4 levels and IgG4^+^ plasma cells in the muscles, mimicking IgG4-RD.

A 73-year-old woman visited our hospital because of proximal muscle weakness of both thighs. Her blood test showed high levels of serum creatinine kinase, aldolase, and IgG4. Magnetic resonance imaging of the thighs showed muscle edema. Needle electromyography showed findings typical of myositis. Histological analysis of her left quadriceps revealed infiltration of IgG4^+^ plasma cells as well as CD8^+^ T cells. Scattered necrotic and regenerating muscle fibers with no specific findings for IgG4-RD (storiform fibrosis and obliterative phlebitis) were typical for PM. We diagnosed her condition as PM and treated her with 40 mg/day of prednisolone that decreased levels of muscle enzymes and improved muscle weakness.

**Conclusion:**

Our case indicated that PM could present with high serum IgG4 levels and IgG4^+^ plasma-cell infiltration, mimicking IgG4-RD. Although the mechanism of IgG4 elevation in such PM is unclear, our case highlights the necessity to recognize that high serum IgG4 levels and IgG4^+^ plasma-cell infiltration in organs are not specific for IgG4-RD.

## 1 Introduction

Polymyositis (PM) is a type of autoimmune, inflammatory myopathy characterized by chronic inflammatory and degenerative changes in the muscles leading to muscle weakness. Mononuclear inflammatory cells infiltrating affected muscles are mainly CD8^+^ T cells rather than B and plasma cells.^[1]^

IgG4-related disease (IgG4-RD) is a rare lymphoproliferative disorder with elevated levels of serum IgG4 and abundant IgG4^+^ plasma cell infiltration of various organs.^[2,3]^ As IgG4-RD has been growingly recognized, cases of other diseases, such as multicentric Castleman disease^[4–8]^ and small vessel vasculitis,^[9]^ presenting with those features have drawn attention because such cases could fulfill the diagnostic criteria for IgG4-RD.^[3,10]^

We herein report the first case of PM with high serum IgG4 levels and IgG4^+^ plasma-cell infiltration of affected muscles, mimicking IgG4-RD. Our case highlights the importance of careful assessment and differential diagnosis in a patient with elevated serum IgG4 levels and tissue IgG4^+^ plasma cell infiltration. An informed consent was obtained from the patient.

## 2 Case presentation

A 71-year-old woman developed muscle pain and weakness in both thighs in 2014. The symptoms gradually progressed, and she was admitted to our hospital for work-up in August 2016.

Physical examinations revealed normal blood pressure of 105/65 mm Hg and body temperature of 36.7°C. Manual muscle testing score of her neck flexor tendons, biceps, triceps, quadriceps, and iliopsoas muscles showed muscle weakness (neck flexor tendons; 2, biceps, triceps, quadriceps, and iliopsoas muscles; bilaterally 4). Gowers’ sign was positive. The findings of ocular, lung, cardiovascular, abdominal, other neurological, joints and skin examination were normal. She had no history of statin treatment.

Laboratory tests revealed elevated levels of serum creatinine kinase (384 IU/L, normal range: 50–170 U/L), aldolase (6.2 IU/L, normal range: 2.7–5.9 IU/L), lactate dehydrogenase (314 U/L, normal range: 120–220 U/L), IgG (2603 mg/dL, normal range: 870–1700 mg/dL), IgA (721 mg/dL, normal range: 93–393 mg/dL), and IgG4 (313 mg/dL, normal range: 4.8–105 mg/dL). While serum erythrocyte sedimentation rate level was elevated (112 mm/h, normal range: 0–15 mm/h), serum C-reactive protein level was within normal range (0.04 mg/dL, normal range: 0–0.35 mg/dL). Other blood tests, including blood counts, serum levels of electrolyte, creatinine, IgE, and blood glucose were within normal range. Anti-nuclear antibody was positive with the titer of 1:1240 (homogeneous and speckled patterns). Antiaminoacyl-tRNA synthetase, anti-RNP, anti-dsDNA antibody, and anti-Sm antibodies were all negative.

**Figure 1:**
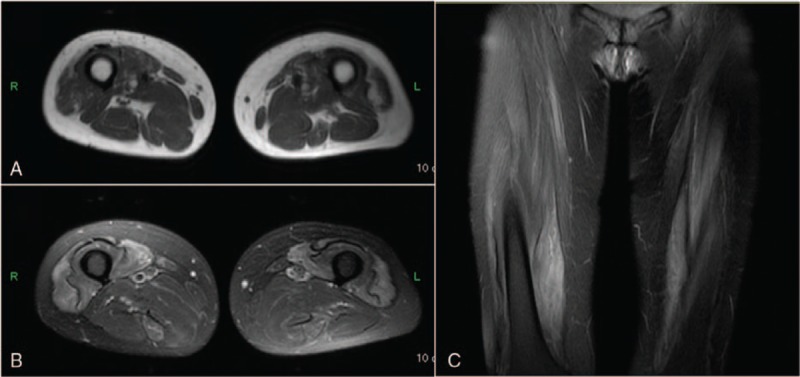
Magnetic resonance imaging of bilateral femoral quadriceps revealed normal intensity on T1-weighted image (A) and high intensity on T2-weighted image (B) and fat-suppressed T2-weighted image (C).

Fat-suppressed T2-weighted magnetic resonance imaging showed high intensity (Fig. 1), and needle electromyography showed spontaneous fibrillations and positive sharp waves in bilateral femoral quadriceps, suggesting the presence of myopathies. Positron-emission tomography-computed tomography demonstrated no abnormal accumulation of fluoro-2-deoxyglucose, or malignant tumor, or typical organ involvement for IgG4-RD, including lacrimal glands, salivary glands, pancreas, bile duct, kidney, aorta, and retroperitoneum.

**Figure 2:**
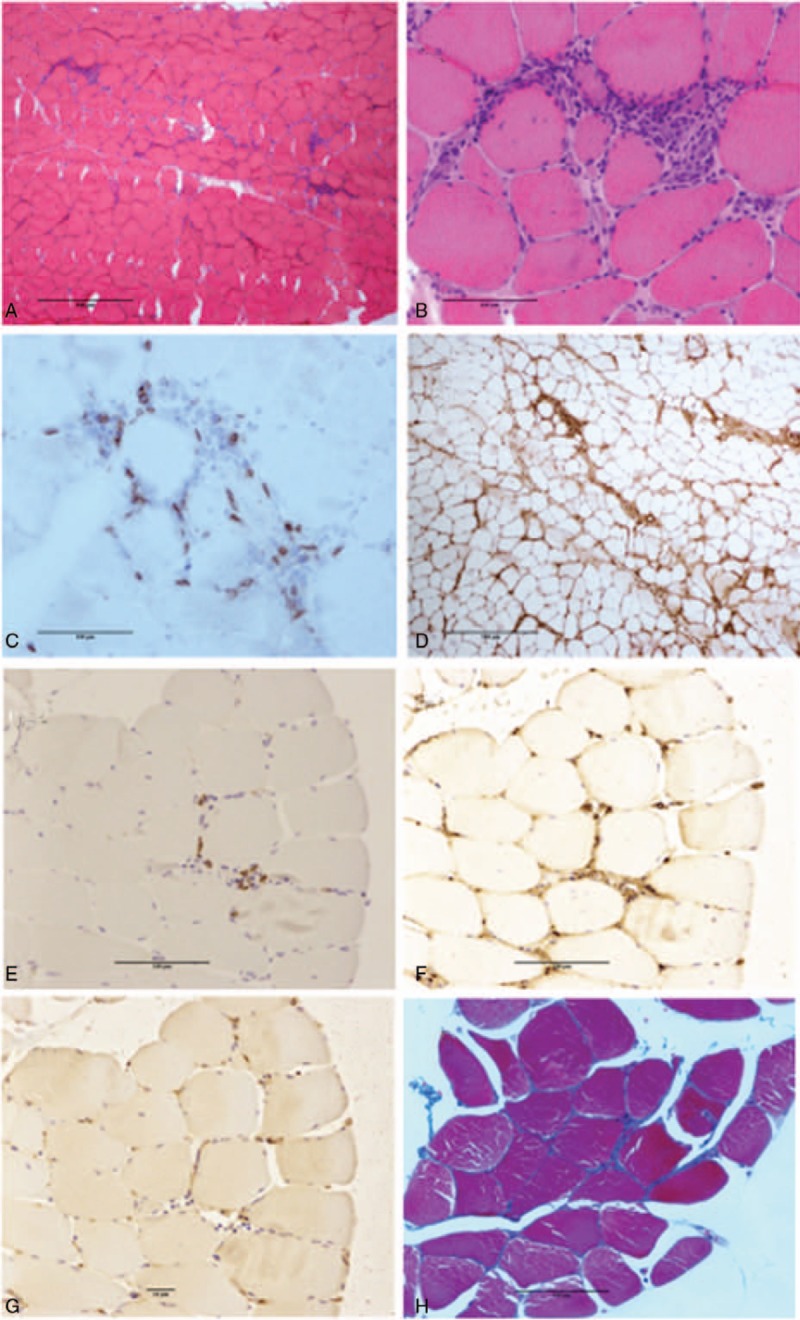
Histopathological findings of muscle biopsy. Hematoxylin–eosin staining revealed presence of mononuclear inflammatory cells that surrounded and invaded muscle fibers and scattering of necrotic tissue and regenerating fibers as well as variations in muscle fiber size (A: low power field; B: high power field). CD8 immunostaining revealed CD8^+^ cell infiltration around the muscle fibers (C). MHC class I antigen was extensively expressed on the surface of almost all muscle fibers (D). CD138 staining revealed CD138^+^ plasma cell infiltration around the muscle fibers (E). IgG (F) and IgG4 (G) immunostaining revealed that the ratio of IgG4^+^/IgG^+^ plasma cells was over 40%. Masson-trichrome staining revealed no storiform fibrosis and obliterative phlebitis (H).

Histological examination of her left quadriceps revealed mononuclear inflammatory cells that surrounded and invaded muscle fibers (Fig. 2A and B). Those inflammatory cells were positive for CD8 (Fig. 2C), and MHC class I antigen was extensively expressed on the surface of almost all muscle fibers (Fig. 2D). Scattered necrotic and regenerating muscle fibers as well as variations in muscle fiber size were characteristic of inflammatory myopathy (Fig. 2A and B). Infiltration of inflammatory cells at perivascular sites and perifascicular atrophy were not observed. While CD138, IgG, and IgG4 immunostaining (Fig. 2E–G) revealed that over 40% of infiltrated IgG positive plasma cells were IgG4^+^ cells, the important findings for IgG4-RD of storiform fibrosis and obliterative phlebitis were not found (Fig. 2H).

We diagnosed her with PM based on the Bohan and Peter classification criteria.^[11,12]^ Although she also met the 2011 comprehensive diagnostic criteria for IgG4-RD,^[3]^ IgG4-RD was unlikely since she had no swelling or masses in systemic organs, the affected lesion was only muscle without typical sites for IgG4-RD, and the findings of muscles showed necrotic and regenerating muscle fibers but not storiform fibrosis and obliterative phlebitis. Treatment with 40 mg/day of prednisolone decreased muscle enzyme levels and improved muscle weakness.

## 3 Discussion

The present case demonstrated that PM could present with high serum levels of IgG4 and infiltration of IgG4^+^ plasma cells into the muscles, mimicking IgG4-RD. Understanding that elevation of serum IgG4 levels and IgG4^+^ plasma-cell infiltration of affected sites could be seen in other diseases as well as IgG4-RD is critically important for appropriate diagnosis and treatment.

Differential diagnosis of IgG4-RD includes a variety of diseases, such as autoimmune diseases, infectious diseases, and malignancies, since those diseases could present with high serum IgG4 and IgG4^+^ plasma-cell infiltration of affected sites; thus, the comprehensive criteria on IgG4-RD^[3]^ and the international statement regarding management and treatment of IgG4-RD^[13]^ have obliged the exclusion of other diseases in diagnosing patients with IgG4-RD. We reviewed cases reported to mimic IgG4-RD published between 2012 and 2016 (Table 1). All patients were diagnosed with other diseases despite the fulfillment of the comprehensive criteria on IgG4-RD.^[3]^ Most were multicentric Castleman disease and polyangiitis, but other diseases like virus infection and malignancies were also reported. Our case is the first report of PM mimicking IgG4-RD, suggesting that such case may be rare but clinically important in respect of disease management, because the response to glucocorticoids, treatment strategy, and prognosis are different between IgG4-RD and PM.^[13,18–20]^

**Table 1:**
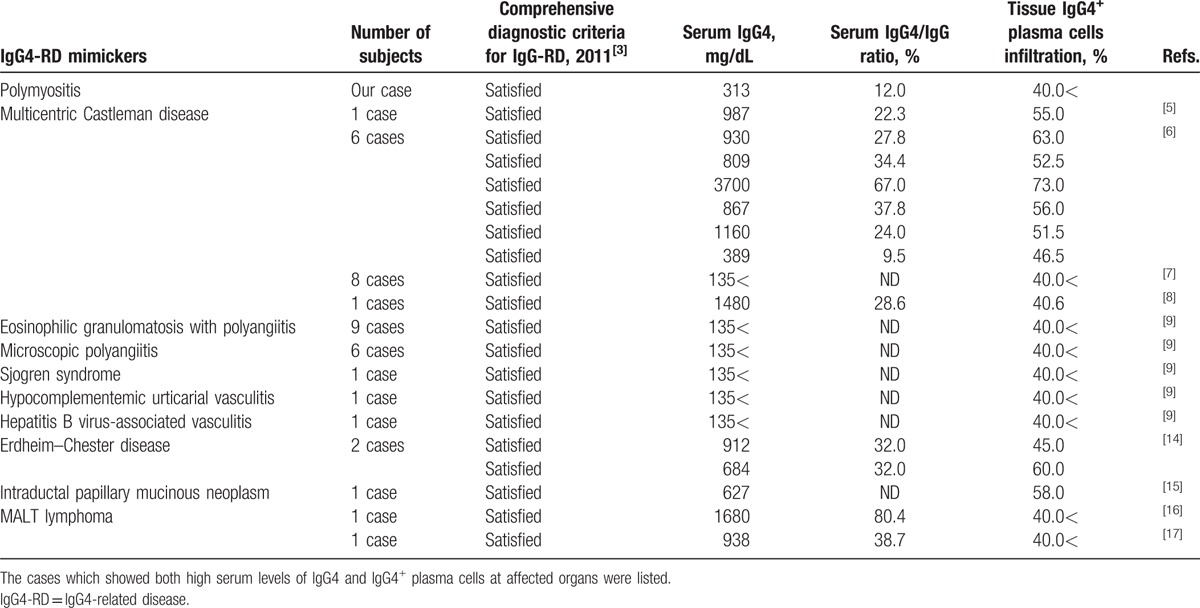
Literature review of IgG4-RD mimickers between 2012 and 2016.

Elevated serum IgG4 levels have been reported in various diseases. Yamamoto et al^[21]^ examined serum IgG4 levels in rheumatic and other diseases, and reported 1 patient with PM among 6 patients with high serum IgG4 levels. While T follicular helper cells induce IgG4 class-switching in IgG4-RD,^[22–26]^ the mechanism of elevation of serum IgG4 levels in PM is unclear. Our case showed not only elevation of IgG4 levels but also elevation of IgA levels, which is not usually found in IgG4-RD, suggesting that the mechanism of IgG4 elevation in PM is different from that of IgG4-RD. We hypothesize that polyclonal elevation of immunoglobulins contributes to IgG4 elevation in PM rather than the specific skewing toward IgG4 class-switching.

Our case showing massive infiltration of plasma cells as well as CD8^+^ T cells at affected muscles has raised another interesting possibility in the pathogenesis of PM. Although T cells are proved important in PM, B cells and plasma cells can also be accountable. The role of B cells and plasma cells in the pathogenesis of PM has been shown by frequent presence of autoantibodies and hypergammaglobulinemia.^[28]^ Moreover, a local in situ differentiation of B cells into mature plasma cells is thought to occur in the muscle tissue of PM as clonal expansion of B cells is present in affected muscle tissues, and significant somatic hypermutation and isotype switching is shown by local immunoglobulin variable region sequences.^[28,29]^ The involvement of B cells in PM is also clinically indicated by favorable responses to rituximab (anti-CD20 monoclonal antibody).^[30,31]^ Taken together, our case warrants further examinations of a role of B cells and plasma cells in PM.

Moderate dose of glucocorticoid is usually effective to IgG4-RD,^[13]^ while a part of patients with PM are refractory,^[18,19]^ and need intravenous immunoglobulins or other immunosuppressive agents like calcineurin inhibitors and cyclophosphamide in addition to high dose glucocorticoids.^[31,32]^ Thus, the differentiation of PM from IgG4-RD is clinically important in the management of the disease.

## 4 Conclusions

We described the first case of PM that presented with elevated serum levels of IgG4 and abundant infiltration of IgG4^+^ plasma cells into affected sites, mimicking IgG4-RD. High serum IgG4 levels and tissue-infiltration of IgG4^+^ plasma cells are indicative but not specific for the diagnosis of IgG4-RD.

## Uncited reference

^[27]^.
